# Energy Metabolism in Exercise-Induced Physiologic Cardiac Hypertrophy

**DOI:** 10.3389/fphar.2020.01133

**Published:** 2020-07-29

**Authors:** Kefa Xiang, Zhen Qin, Huimin Zhang, Xia Liu

**Affiliations:** Department of Clinical Pharmacy, School of Pharmacy, Second Military Medical University, Shanghai, China

**Keywords:** energy metabolism, exercise, physiologic cardiac hypertrophy, signaling molecules, regulatory mechanism

## Abstract

Physiologic hypertrophy of the heart preserves or enhances systolic function without interstitial fibrosis or cell death. As a unique form of physiological stress, regular exercise training can trigger the adaptation of cardiac muscle to cause physiological hypertrophy, partly due to its ability to improve cardiac metabolism. In heart failure (HF), cardiac dysfunction is closely associated with early initiation of maladaptive metabolic remodeling. A large amount of clinical and experimental evidence shows that metabolic homeostasis plays an important role in exercise training, which is conducive to the treatment and recovery of cardiovascular diseases. Potential mechanistic targets for modulation of cardiac metabolism have become a hot topic at present. Thus, exploring the energy metabolism mechanism in exercise-induced physiologic cardiac hypertrophy may produce new therapeutic targets, which will be helpful to design novel effective strategies. In this review, we summarize the changes of myocardial metabolism (fatty acid metabolism, carbohydrate metabolism, and mitochondrial adaptation), metabolically-related signaling molecules, and probable regulatory mechanism of energy metabolism during exercise-induced physiological cardiac hypertrophy.

## Introduction

The health benefits of exercise are indisputable. Exercise not only reduces cardiac risk factors and improves cardiac function, but also reduces mortality and morbidity from various cardiovascular diseases (Lavie et al., 2015; Tao et al., 2015; Ostman et al., 2017; Acosta-Manzano et al., 2020; Bersaoui et al., 2020). Some researchers have demonstrated the cardiac protective effect of regular exercise on patients with cardiovascular disease (Ellison et al., 2012), and endurance training can improve cardiac performance by transforming pathological cardiac hypertrophy into a physiological state (Garciarena et al., 2009). However, the exact mechanism of this cardiac adaptation is not fully understood.

**Figure 1:**
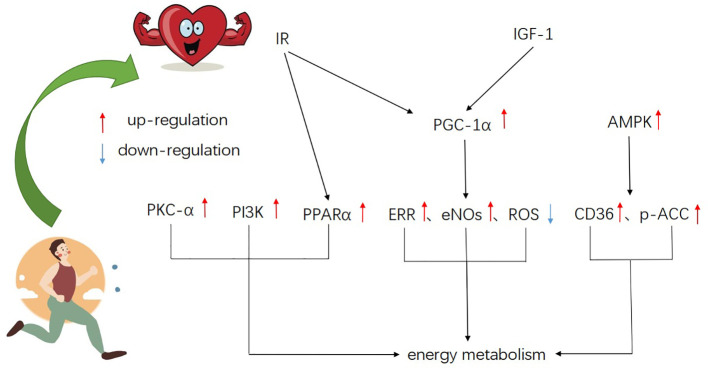
A schematic of the major signaling molecules of metabolism in exercise-induced physiological myocardial hypertrophy, showing integration and cross-talk of various pathways.

**Figure 2:**
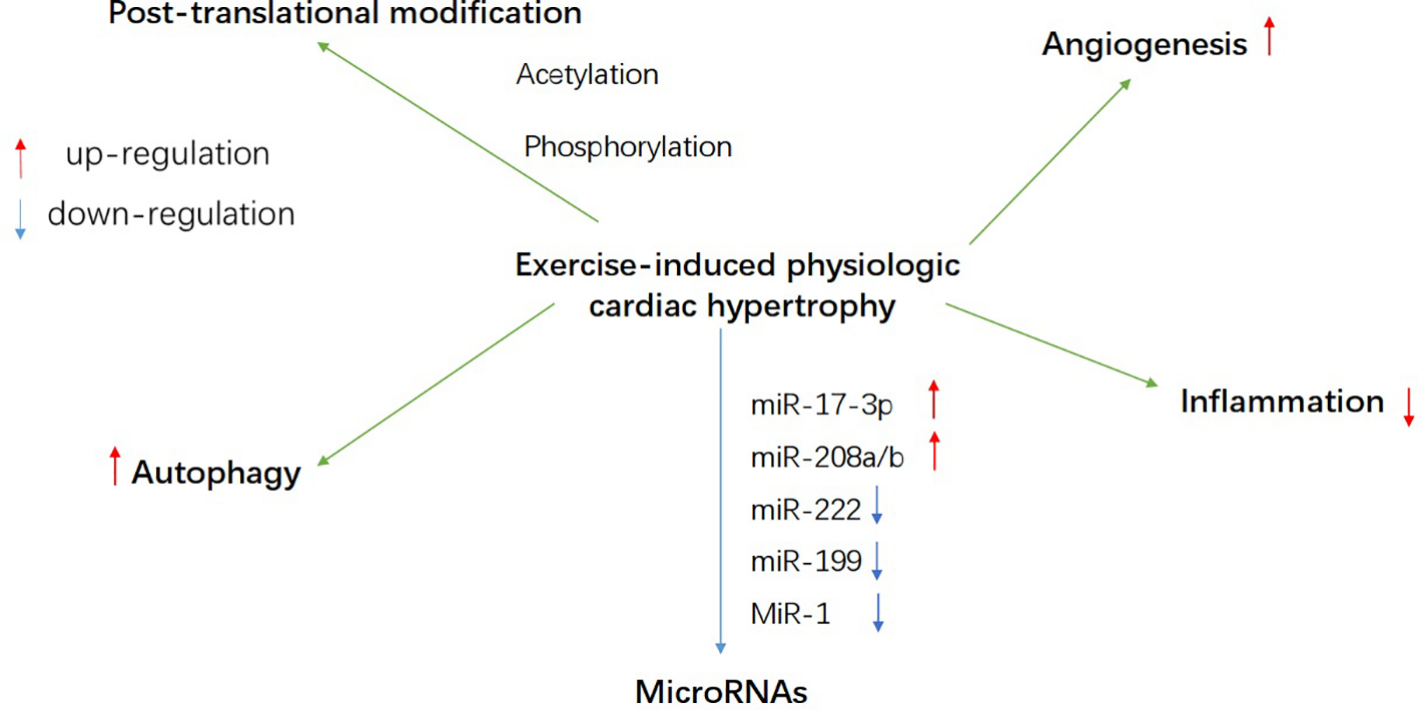
A schematic overview of probable regulatory mechanism of energy metabolism during exercise-induced heart growth, such as autophagy, post-translational modification, microRNAs, angiogenesis, and inflammation.

Notably, exercise can induce physiological cardiac hypertrophy (Ellison et al., 2012; Xiao et al., 2014; Xiao et al., 2016). In this state, myocardial hypertrophy has normal or enhanced contractile function, considered adaptive, and is not a risk factor for heart failure (Shimizu and Minamino, 2016). Physiological cardiac hypertrophy is characterized by mild heart growth, its mass is usually 10%‑20% higher than that of normalized heart (Maillet et al., 2013). The physiological hypertrophy of myocardium induced by exercise training is different from pathological hypertrophy at the stimulation mode, structure, and molecular level (McMullen and Jennings, 2007). In a study of myocardial gene expression profile in rats, it was shown that during exercise-induced physiological myocardial hypertrophy, the glucose signal of cardiomyocytes was significantly different, while there was no significant change in pathological hypertrophy (Kong et al., 2005). A large number of studies have proved that the metabolic coordination is an essential condition for myocardial adaptive growth (Turpeinen et al., 1996; Peterzan et al., 2017; Fulghum and Hill, 2018; Gibb and Hill, 2018; Heallen et al., 2020), but it is not clear whether the regulation of myocardial energy metabolism can reverse myocardial remodeling and improve myocardial function. Therefore, the study of energy metabolism in exercise-induced physiological cardiac hypertrophy is beneficial to the exploration of exercise-induced cardiac adaptation and might be a unique research perspective for interventions in heart failure and other cardiovascular diseases. This review describes the variation of energy metabolism in exercise-induced physiological myocardial hypertrophy from three aspects: fatty acid metabolism, carbohydrate metabolism, and mitochondrial adaptation, and summarizes the related signal molecules (**Figure 1**) and possible regulatory pathways of this energy metabolism (**Figure 2**).

## Changes of Myocardial Metabolism in Exercise-Induced Physiologic Cardiac Hypertrophy

The heart has to keep contracting to provide the body with the oxygen and nutrients it needs. Under normal physiological conditions, due to the small storage of high energy phosphate in cardiac myocytes, normal heart function depends on the tight coupling of intracellular ATP production and myocardial contraction (Kolwicz et al., 2013), and ATP is mainly derived from the catabolism of glucose and fatty acids. Glucose oxidation is a key source of myocardial ATP. In healthy adults, the heart obtains about 50%‑70% of myocardial acetyl CoA derived ATP from fatty acids (Lopaschuk et al., 1994). When endogenous fatty acid supply is reduced, intracellular triacylglycerol hydrolyzes the fatty acid back through specific lipases including adipose triglyceride lipase (ATGL) (Haemmerle et al., 2011; Kienesberger et al., 2012) for fatty acid oxidation and subsequent ATP generation. The efficient absorption and recycling of fatty acids by the heart is the key to ensuring ATP supply and systolic function (Kim and Dyck, 2016), and glycolysis produces less than 10% of total ATP in healthy hearts (Lygate et al., 2013). The main pathway for ATP resynthesis is mitochondrial oxidative phosphorylation (>98%), which is driven by the reduction equivalent NADH and FADH2 produced by fatty acid oxidation, pyruvate oxidation, and Krebs cycle. In a healthy heart, the hydrolysis rate of ATP matches the rate of ATP resynthesis, and the tissue content of ATP is very constant, even though the conversion rate of ATP is greatly raised (Stanley and Chandler, 2002). The elevated acute load during exercise has a strong effect on myocardial metabolism (Gibala et al., 1998). In the heart, exercise boosts contractility and oxygen consumption, which is 10 times higher than the resting rate (Olver et al., 2015). Although the heart can take advantage of substrates including carbohydrates, lipids, amino acids, and ketone bodies, to provide energy, while its substrate preference vary under both physiological and pathological stress (Ritterhoff and Tian, 2017). The changes of substrate utilization and mitochondrial adaptation induced by exercise in physiological cardiomyocytes are effective guarantees to maintain the normal myocardial cells function.

### Fatty Acid Metabolism

Exercise heightens fat catabolism in adipose tissue and increases triglycerides and free fatty acids in plasma, thus promoting the utilization of fatty acids. It was found that the myocardial hypertrophy of female mice after exercise was significantly enhanced compared with that of male mice, which may be related to the heightened level of free fatty acid in plasma caused by exercise (Foryst-Ludwig et al., 2011). During exercise, hormone-mediated lipid interpretation of fatty acid metabolites in adipose tissue during exercise potentially promotes cardiac physiological growth by activating G-protein-coupled receptors (GPCRs), Akt, or nuclear receptors (Foryst-Ludwig et al., 2015). Not only did free fatty acids increase rapidly with exercise (Frandsen et al., 2019), but they also seemed to remain elevated during exercise adaptation, and the elevated levels of free fatty acids in plasma were considered sufficient to promote cardiac fat catabolism (Pels et al., 1985; Monleon et al., 2014). The uptake of free fatty acids by cardiomyocytes was achieved through plasma membrane transporters, fatty acid translocases (FAT/CD36), fatty acid transporters (FATP), and to a lesser extent, transmembrane diffusion. CD36 deficiency will result in defective fatty acid uptake (Tanaka et al., 1997; Abumrad and Goldberg, 2016). In exercise-induced physiological myocardial hypertrophy, fatty acid and glucose oxidation are enhanced, accompanied by increasing gene expression of encoding fatty acid transporters, fatty acid binding proteins, and lipid metabolic pathways (Strøm et al., 2005; Dobrzyn et al., 2013; Nakamura and Sadoshima, 2018). After fatty acid uptake and conjugation with acetyl CoA (FA-CoA), FA-CoA enters the mitochondria, *via* the carnitine acyl transferase shuttle (CPT-1 and CPT-2) (Abel and Doenst, 2011). CPT-I and CPT-II are responsible for the input of mitochondrial fatty acids and control the rate-limiting steps of the mitochondrial fatty acid oxidation pathway (Lehman et al., 2000). The up-regulated expressions of CPT-I and CPT-II were found in a mice model of exercise training and CPT-II mRNA expression was significantly heightened in exercise-induced physiologic myocardial hypertrophy (Iemitsu et al., 2003). Acetyl coenzyme A carboxylase (ACC) is a key enzyme in fatty acid synthesis. An increase in ACC phosphorylation was observed in cardiomyocytes during moderate exercise training, including the phosphorylation of ACC-1 (265 kDa) and ACC-2 (280 kDa) (Coven et al., 2003). It is possible that ACC phosphorylation reduces the generation of malonyl coenzyme A, thereby indirectly lightening its inhibition of CPT-I activity, increasing the level of free fatty acids in plasma (Awan and Saggerson, 1993; Abu-Elheiga et al., 2001), and playing an important role in regulating fatty acid oxidation (Lopaschuk et al., 1994).

### Carbohydrate Metabolism

Glucose uptake in the heart was found to be transversely distributed, and the subendocardial layer is about 30% higher than the epicardial layer. The altered transmural distribution of glucose uptake after exercise probably reflects the metabolic adaptation of different myocardial layers to the physiologic growth of cardiomyocytes induced by exercise (Takala et al., 1983; Kainulainen et al., 1985; Kainulainen et al., 1989). It was found that exercise training elevated GLUT4 mRNA levels of left ventricle in rats (Vettor et al., 2014). The myocardial glucose uptake was significantly higher in the exercise group than that in the rest group (Gertz et al., 1988; Kemppainen et al., 2002). In addition, experimental studies have demonstrated that the elevation of catecholamine and intracellular calcium concentration can raise GLUT4 translocation in the heart (Rattigan et al., 1991), while exercise can activate the sympathetic nerve, elevate catecholamine levels in the body, strengthen calcium concentration and myocardial contractility in cardiomyocytes. These mechanisms may all play a role in promoting glucose uptake during exercise. At the same time, studies have indicated that the glucose utilization rate of cardiomyocytes in the exercise group is significantly lower than that in the control group (Monleon et al., 2014). At present, only pyruvate kinase activity has been found to be elevated in exercise-adapted rat hearts (York et al., 1975; Stuewe et al., 2000), but little is known about the effect of exercise on rate-limiting enzymes in glycolysis pathway. Glycolysis, aerobic oxidation of glucose and glycogen synthesis, were all enhanced *in vitro* perfusion hearts of exercise-adapted mice (Riehle et al., 2014). However, in exercise-adapted rats, basal myocardial glycolysis was reduced despite increased myocardial glucose and palmitate oxidation (Burelle et al., 2004). Differences between the two studies may be due to differences in animal models (such as rodent species, exercise type) or differences in cardiac perfusion regiments (matrix levels, hormone addition). It was found that the glycogen accumulation in the perfused heart of exercise training adapted mice (Riehle et al., 2014) may be due to the increased glucose uptake stimulated by insulin (Jensen and Richter, 2012). The cardiac glycogen is a potential source of myocardial energy (Aguiar et al., 2017), and glycogen resynthesis after exercise contributes to glucose homeostasis. Recent studies have manifested that cardiac-specific expression of a kinase-deficient 6-phosphofructo-2-kinase/fructose-2,6-bisphosphatase transgene lowered glycolytic rate and regulated the expression of genes known to promote cardiac growth (Boström et al., 2010; Bezzerides et al., 2016; Gibb et al., 2017). These researches further illustrated that depressed glycolytic activity appeared to be important in exercise-induced physiological cardiac hypertrophy. These experimental results showed that although glucose uptake, aerobic oxidation, and glycogen synthesis are all prolonged in exercise-induced hypertrophic myocardium, cardiomyocytes still give priority to fatty acid oxidation to provide energy. Metabolic adaptation maintains the balance between glucose and fatty acid catabolism on the premise of improving the efficiency of cardiac energy production.

### Mitochondrial Adaptation

The role of mitochondria in physiological myocardial hypertrophy cannot be ignored. Exercise training promoted mitochondrial biosynthesis in the heart (Vettor et al., 2014), which was related to exercise-induced cardiac hypertrophy (White et al., 1987; Rimbaud et al., 2009a; Abel and Doenst, 2011). A recent study showed that a single exhausting exercise in untrained or trained rats resulted in increasing levels of cytochrome C, Caspase3, and mitochondrial DNA deletion (Huang et al., 2009), suggesting that mitochondrial damage may be an indirect signal to activate mitochondrial biogenesis. It was found that mitochondrial DNA synthesis, electron transport chain related enzyme activity and citrate synthase activity all increased in the hearts of swimming training mice (Riehle et al., 2014). The energy consumption of the heart during exercise is several times higher than that of the resting state, which is related to the increasing of oxygen consumption and mitochondrial ATP production rate. Meanwhile, the myocardial mitochondrial ATP production rate must be highly matched with the ATP decomposition rate. Swimming training induced the enhancement of mitochondrial respiration and ATP production in physiological myocardial hypertrophy of mice (Ascensão et al., 2011), and some experiments proved that the mitochondrial respiration of isolated myocardium of mice also increased after exercise training, which was consistent with the change of gene expression of fatty acid utilization (O’Neill et al., 2007). In addition, exercise training promotes myocardial physiological hypertrophy while carrying out adaptive remodeling of mitochondria (Bo et al., 2010). Eight weeks of exercise conditioned training increased the number of myocardial mitochondria, especially the smaller ones (Dworatzek et al., 2014). In mice, exercise strongly promotes the division of myocardial mitochondria, thereby enhancing the function of mitochondria; these mitochondrial changes occur in a manner dependent on adrenergic signals (Coronado et al., 2018). In cardiomyocytes, mitochondria are considered to be the main source of reactive oxygen species. Under normal/basic conditions, ROS is produced as a by-product of mitochondrial electron transfer activity and is buffered by antioxidant systems. Exercise-induced reactive oxygen species may have beneficial effects on heart growth (Sadoshima, 2006; Alleman et al., 2014; Schieber and Chandel, 2014), probably because ROS, a second messenger to change redox sensitive enzymes, contributes to exercise-induced mitochondrial adaptive signal transduction.

The above studies showed that the adjustment of myocardial energy metabolism contributes to exercise-induced physiological myocardial hypertrophy. Although fatty acids seem to favor energy production, the heart has the ability to respond quickly to variation in matrix availability, ensuring that ATP production continues to meet its energy needs (Taegtmeyer et al., 2004; Hue and Taegtmeyer, 2009; Smith et al., 2018). Therefore, exercise training is related to the regulation of fatty acid, glucose metabolism and mitochondrial adaptation, which may further promote the coordination of myocardial metabolic flexibility and myocyte physiological hypertrophy. However, the specific mechanism is still not well understood and needs further study.

## The Signaling Molecules of Metabolism in Exercise-Induced Physiological Myocardial Hypertrophy

### PGC-1α

Peroxisome proliferator-activated receptor-γ coactivator-1α (PGC-1α) is a transcriptional coactivator initially identified as a cold-inducing factor involved in the mitochondrial biogenesis of brown adipocytes (Puigserver et al., 1998). PGC-1α is required for the normal reserve of fatty acid oxidation (FAO) (Lehman et al., 2008; Peterzan et al., 2017), and PGC-1β for the normal expression of OXPHOS gene (Vianna et al., 2006). PGC-1α/β promotes coordination of gene transcription, mitochondrial biosynthesis, and growth signal at various levels of oxidative metabolism (Rowe et al., 2010). The expression of PGC-1α increases in exercise-induced myocardial hypertrophy, which promotes the production of mitochondria and the ability of fatty acid oxidation (FAO) (O’Neill et al., 2007; Watson et al., 2007; Riehle et al., 2014). The cardiac function of adult PGC-1α/β-deficient mice does not show obvious abnormalities, but the expression of genes related to the mitochondrial energy transmission pathway (including FAO, tricarboxylic acid cycle, ETC/OXPHOS) in the gene expression profile is significantly and widely down-regulated (Martin et al., 2014). It is probably that PGC-1α/β is not necessary to maintain mitochondrial density and cardiac function in the basal state (Vega et al., 2015), but in the process of exercise-induced physiological myocardial growth, it is necessary to maintain the high volume respiratory function of hypertrophic myocardial mitochondria by driving the expression of genes related to mitochondrial energy conduction and ATP synthesis pathway. PGC-1α also promotes a broader mitochondrial biological response through interaction with estrogen-related receptors (ERR) and nuclear receptor factor 1 (NRF-1) (Scarpulla et al., 2012). Studies have demonstrated that, ERR directly activates mitochondrial energy metabolism involving TCA cycle, electron transfer, and oxidative phosphorylation in cardiomyocytes (Dufour et al., 2007). In the exercise training experiment, endothelial nitric oxide synthase (eNOS) in left ventricular murine tissue (Kojda et al., 2001; Zhang et al., 2007) elevates the gene expression of PGC-1α (Vettor et al., 2014). In addition, it is also found that PGC-1α plays an important role in protecting cardiomyocytes from ROS-mediated injury. In the hearts of PGC-1α knockout mice, the basic mRNA expression levels of ROS detoxifying enzymes such as cytoplasmic copper/zinc-SOD1, mitochondrial Mn-SOD, and peroxisome catalase were significantly reduced (St-Pierre et al., 2006). However, whether ERR, eNOS and ROS play a role in cardiac exercise adaptation by affecting the expression of PGC-1α needs further study.

### PPARα

The content of PPARα and PPARβ in cardiomyocytes are the highest, while the expression level of PPARγ is low (Vega et al., 2015). Although both PPARα and PPARβ were initially considered to be regulators of peroxisome β-oxidation (Issemann and Green, 1990), it was later found that only the activation of PPARα in cardiomyocytes regulated the uptake, oxidation and storage of fatty acids in the heart (Gulick et al., 1994; Brandt et al., 1998; Mascaró et al., 1998). Research finds that Cn works by activating PPARα to promote mitochondrial energy production and myocardial growth (White et al., 1987; Turpeinen et al., 1996). Initially, the increasing expression of myocardial peroxisome proliferator-activated receptor (PPARα) was detected during treadmill training in mice (Dobrzyn et al., 2013). Later, it was found that the level of PPARα also increased in exercise-induced physiologic hypertrophic cardiomyocytes (Youtz et al., 2014). In addition, some experiments found that gene expression of PPARα was reduced in pathological cardiac hypertrophy, suggesting that upregulation of PPAR-α expression may limit pathological cardiac hypertrophy (Barger et al., 2000). However, another study explained that the elevating levels of PPARα may be the result of physiological hypertrophy (Rimbaud et al., 2009b). These results further illustrated that PPARα may play an important role in inhibiting the transformation from physiological growth to pathological hypertrophy of cardiomyocytes.

### AMPK

In skeletal muscle, AMPK is activated during exercise to increase metabolism (Winder and Hardie, 1996; Winder et al., 1997). AMPK also acts as an important energy sensor and metabolic regulator in the heart (Bairwa et al., 2016). When activated by increasing energy demand, its role is to boost the intake and catabolism of fatty acids (Hardie, 2004). The increase in myocardial stromal metabolism during exercise can be explained by the activation of AMPK (Coven et al., 2003). The activation of AMPK can enhance the uptake of long-chain fatty acids (LCFA) in wild-type cardiomyocytes (Habets et al., 2007) but not the uptake in CD36 knockout cardiomyocytes (Samovski et al., 2015), promote the transport of CD36 to the plasma membrane (Luiken et al., 2003; Kim and Dyck, 2016), indicating that AMPK promotes the CD36 expression and transport to raise fatty acid supply. In addition, AMPK plays a role in myocardial metabolism by phosphorylating ACC, a key enzyme that activate fatty acid metabolism (Nagendran et al., 2013; Hardie et al., 2016). It was found that CD36 expression and ACC phosphorylation are up-regulated in physiologically hypertrophic myocardium induced by exercise training (Strøm et al., 2005; Dolinsky and Dyck, 2006). Thus, AMPK may strengthen the energy supply of exercise-induced myocardial hypertrophy through interaction with CD36 and ACC.

### PI3K

Mitochondrial-associated factor phosphatidylinositol 3 kinase (PI3K) signaling and its downstream effectors Akt and GSK-3β promote myocardial physiological hypertrophy growth and maintain normal cardiac function (McMullen et al., 2003). Although PI3K inhibition can reduce the size of the heart and prevent the adaptation of mitochondria to physiological hypertrophy (Shioi et al., 2000). However, the downregulation of the effector Akt by PI3K is not necessary for mitochondria to adapt to cardiac hypertrophy, suggesting that there is an independent PI3K signaling pathway in the induction of physiological hypertrophy and may be related to the regulation of mitochondrial metabolism (O’Neill et al., 2007). It was found that the repolarization of K^+^ current amplitude of ventricular myocytes in PI3K cultured group and swimming training induced physiological cardiac hypertrophy group are both higher than that of the wild type, while the expression of transcripts encoding K^+^, Ca^2+^, and other ion channel subunits is elevated, which is parallel to the increased cardiomyocyte size and total cellular RNA expression. It is suggested that the steady-state regulation of physiological myocardial hypertrophy and excitability induced by exercise may be related to myocardial PI3Kα signal (Yang et al., 2010), and the normal maintenance of myocardial electrical function is inseparable from energy. Therefore, PI3K may be related to the energy regulation of maintaining electrical excitation homeostasis in exercise-induced physiological myocardial hypertrophy.

### IGF-1R and IR

Insulin-like growth factor-1 receptor (IGF-1R) has been proved to be an important condition for inducing myocardial physiological growth in mice (McMullen et al., 2004; Kim et al., 2008). In heart-specific IR knockout mice, the expression of PPARα decreased, the level of PGC-1α did not change. Meanwhile, the mitochondrial respiration and ATP synthesis rate were impaired (Boudina et al., 2009). The deletion of insulin receptor substrate (IRS), which is necessary for IGF-1R and IR signal transduction, preventing the activation of PGC-1α, but increasing the capacity of mitochondria after exercise (Riehle et al., 2014). These results provide evidence that the coordination of growth procedures and metabolic reprogramming may occur in insulin-like growth factor-1 and insulin triggered signaling pathways.

### PKC-α

Experiments with swimming-trained mice have shown that activation of PKC-α suppresses apoptosis, promotes the growth of physiological cardiomyocytes and improves cardiac function (Naskar et al., 2014). Excessive activation of PKC-α in the liver affects glucose and fatty acid metabolism. In myocardium, PKC-α activation protects myocardium by improving cardiac mitochondrial function (Nowak and Bakajsova, 2013). These related researches of PKC-α indicated that the role of PKC-α in exercise-induced physiological hypertrophy may be associated with the regulation of metabolism.

## The Probable Regulatory Mechanism of Energy Metabolism During Exercise-Induced Heart Growth

### Autophagy

The recycling of cell components by autophagy has become an important process of adaptive response to exercise (Halling and Pilegaard, 2017). Many beneficial effects of exercise may be related to autophagy, especially physiological myocardial hypertrophy caused by exercise. It was found that the activity of autophagy increased significantly in the heart of exercise-induced physiologically hypertrophic rats (Qi et al., 2020). Some previous studies have shown that autophagy/mitophagy plays an important role in the adaptation of skeletal muscle to endurance exercise and interacts with mitochondrial organisms (He et al., 2012; Vainshtein et al., 2015; Ju et al., 2016). A team found that mitochondrial autophagy-related proteins Beclin1, LC3, and Bnip3 are significantly up-regulated during acute exercise (Li et al., 2016a). Therefore, there may be a certain interaction between autophagy and metabolism in exercise-induced physiological hypertrophic cardiomyocytes.

### Post-Translational Modification

Post-translational modification refers to the process of covalent processing of the translated proteins. There are more than 20 post-translational modification processes in eukaryotic cells, such as acetylation, phosphorylation, ubiquitination, and methylation. The mitochondrial proteome consists of approximately 1100 to 1500 proteins, most of which are encoded by nuclear DNA and transferred to mitochondria after post-translational modification (Zhou et al., 2013). Studies have found that metabolic adjustment of myocardium during exercise may be related to ACC phosphorylation in fatty acid metabolism (Coven et al., 2003). By activating PKC-δ and simultaneously inhibiting PKC-α phosphorylation, the PKC subtype is reversed, resulting in impaired cardiac function during physiological hypertrophy (Naskar et al., 2014). In addition, a research shows that the protein O-GlcNAcylization in physiologically growing cardiomyocytes in swimming trained mice is reduced as a whole compared to sedentary mice (Belke, 2011). The O-junction of monosaccharide β-N-acetyl-glucosamine (O-GlcNAcylization) is a post-translational modification on serine and threonine residues, which is an important mechanism for regulating various cellular processes (Mailleux et al., 2016). Another study compared the low and high running ability of rats. The author mentioned the difference in the level of cardiac O-GlcNAcylization of several mitochondrial proteins, among which the O-GlcNAc levels of complex I and complex IV proteins in the low-volume group are higher than high capacity group (Johnsen et al., 2013). Therefore, O-GlcNAcylization may play a role in mitochondrial adaptation to exercise-induced cardiac hypertrophy. Meanwhile, in the physiological cardiac hypertrophy induced by aerobic exercise, PPARβ and histone deacetylase (HDAC) I and II were found to be elevated, accompanied by the interaction between metabolism and epigenetic genes (Soci et al., 2016). Deacetylase (HDAC) affects the acetylation of histones, and exercise training can accelerate the up-regulation of HDAC gene expression and increase the acetylation of histones in cardiomyocytes (Medford et al., 2013; Konhilas et al., 2015; Bernardo et al., 2018). In addition, myocardium-specific histone deacetylase HDAC3 (a member of HDAC I) plays a unique role in maintaining cardiac function and modulating fatty acid metabolism by regulating histone acetylation in the promoter region of myocardial energy gene (Montgomery et al., 2008). These studies indicated that posttranslational modification may play a complementary role in regulating cardiac growth and myocardial energy metabolism.

### MicroRNAs

MicroRNAs are small non-coding RNA molecules with a length of about 22 nucleotides, which activate the post-transcriptional gene expression by binding to target messenger RNA to promote its degradation. Some animal and human studies have shown that the levels of microRNAs in exercise-induced cardiac hypertrophy have been altered (**Table 1**). Among them, the expressions of miR-17-3p, miR-30, miR-21, miR-27a/b, miR-144, miR-145 (Wang et al., 2018), miR-29 (Soci et al., 2011), miR-19b, miR-208b, and miR-133b (Ramasamy et al., 2015) were up-regulated. MiR-1, miR-133, miR-124, miR-143 (Wang et al., 2018), miR-341 (Martinelli et al., 2014), miR-100, miR-22, miR-99b, miR-181a, miR-191a (Ramasamy et al., 2015), miR-214 (Melo et al., 2015), miR-199, miR-222 (Schüttler et al., 2019), miR-26b-5p, miR-204-5p, and miR-497-3p (Qi et al., 2020) were down-regulated. MicroRNAs regulate gene expression in hypertrophic myocardium induced by exercise training, in which miR-17-3p and miR-222 may induce cardiomyocyte metabolism and mitochondrial adaptation (Schüttler et al., 2019). Furthermore, inhibition of endogenous miR-199 inducing physiological myocardial hypertrophy may be related to up-regulation of PGC-1α mRNA expression (Li et al., 2016b). MiR-1 suppresses the translation of MCU (a kind of mitochondrial calcium transporter), reduces the uptake of mitochondrial Ca^2+^ in cardiomyocyte, and increases the production of metabolic energy (Zaglia et al., 2017). Inhibition of miR-208b increases the expression of epigenetic target proteins, which may stimulate the interaction between metabolism and cell growth (Soci et al., 2016). In addition, in a study on the relationship between miRNAs and exercise-induced physiological cardiac hypertrophy, KEGG pathway analysis showed that 12 up-regulated miRNAs are associated with fatty acid degradation, fatty acid metabolism, and fatty acid elongation (Xu et al., 2017). These researches indicated that microRNAs may have a certain effect on metabolism in maintaining exercise-induced physiological myocardial hypertrophy. However, the roles of lncRNAs and circRNAs in physiological cardiac hypertrophy have not been reported (Wang et al., 2020).

**Table 1:** Overview of microRNA levels altered in exercise-induced physiologic cardiac hypertrophy and their cellular targets.

MicroRNA	Cellular Target	Animal Model and Exercise Modality	References
miR-17-3p	TIMP3, PTEN	Mice, swimming exercise	(Wang et al., 2018)
miR-30	P53, Drp-1	Mice, treadmill running	(Wang et al., 2018)
miR-21	PDCD4	Mice, treadmill running	(Wang et al., 2018)
miR-27a/b	ACE	Rats, swimming exercise	(Wang et al., 2018)
miR-144	PTEN	Rats, swimming exercise	(Wang et al., 2018)
miR-145	TSC2	Rats, swimming exercise	(Wang et al., 2018)
miR-29	collagen	Rats, swimming exercise	(Soci et al., 2011)
miR-19b	PTEN, MuRF, Bcl2, Atrogin-1, αCryB	Rats, swimming exercise	(Ramasamy et al., 2015)
miR-208b	THRAP1, Myostatin	Rats, swimming exercise	(Ramasamy et al., 2015; Soci et al., 2016)
miR-133b	CyclinD, NelfA, RhoA, Ccd42	Rats, swimming exercise	(Ramasamy et al., 2015)
miR-100	IGF1R, Akt, mTOR	Rats, swimming exercise	(Ramasamy et al., 2015)
miR-22	CDK6, Sir1, Sp1	Rats, swimming exercise	(Ramasamy et al., 2015)
miR-99b	IGF1R, Akt, mTOR	Rats, swimming exercise	(Ramasamy et al., 2015)
miR-181a	MAPK1, TNFα, GATA4	Rats, swimming exercise	(Ramasamy et al., 2015)
miR-191a	Egr1, Cd4, Casp4, SOCS4, p53	Rats, swimming exercise	(Ramasamy et al., 2015)
miR-1	Bcl-2	Mice, treadmill running	(Zaglia et al., 2017; Wang et al., 2018).
miR-133	Calcineurin, PI3K/Akt signaling	Rats, swimming exercise	(Wang et al., 2018)
miR-124	PI3K	Rats, swimming exercise	(Wang et al., 2018)
miR-143	ACE2	Rats, swimming exercise	(Wang et al., 2018)
miR-341	c-Myb	Mice, treadmill running	(Martinelli et al., 2014)
miR-199	PGC1α	Mice, treadmill running	(Li et al., 2016b; Schüttler et al., 2019)
miR-222	P27, Hipk1, Hmbox1	Mice, swimming exercise	(Schüttler et al., 2019)
miR-214	SERCA2a	Rats, leg flexing exercise	(Melo et al., 2015)
miR-26b-5p	LC3B, Beclin1, SQSTM1	Rats, swimming exercise	(Qi et al., 2020)
miR204-5p	LC3B, Beclin1, SQSTM1	Rats, swimming exercise	(Qi et al., 2020)
miR-497-3p	LC3B, Beclin1, SQSTM1	Rats, swimming exercise	(Qi et al., 2020)

### Angiogenesis

VEGFs are main regulators of myocardial angiogenesis. Some studies have found that inhibition of VEGFs signal transduction leads to capillary thinning and early heart failure (Izumiya et al., 2006; Sano et al., 2007), indicating that cardiomyocytes may produce angiogenic factors to maintain capillary density, oxygen supply, and function. The number of myocardial capillaries increased significantly in physiological myocardial hypertrophy (Anversa et al., 1983; Oka et al., 2014), while the capillary density was attenuated in pathological myocardial hypertrophy (Anversa and Capasso, 1991; Beltrami et al., 1994), suggesting that the number of myocardial capillaries is controlled by the myocardium, and the decrease in capillary density may cause hypoxia or even contraction dysfunction. Therefore, the growth of physiologically hypertrophic cardiomyocytes induced by exercise may be related to angiogenesis providing a good oxygen supply for myocardial metabolism.

### Inflammation

There are total 59 genetic variation in the study of physiological cardiac hypertrophy in swimming mice or treadmill rats, only two genes (Cd74 and Col3a1) are changed in both mice and rat models. The immune-function related gene Col3a1 was down-regulated in response to exercise in mice (1.6, 3.1-fold) and rats (1.8-fold) but up-regulated in most pathological cardiac hypertrophy models (Galindo et al., 2009). In the study of cardiac cell RNA sequencing, it was found that one of the most significant difference in the physiologically hypertrophic myocardium induced by swimming training was the severe downregulation of the autoimmunity pathway, accompanied by the obvious selective splice of exon variants (AS) (Song et al., 2012). In addition, in a study using high-density oligonucleotide microarray to detect myocardial gene expression profiles in physiologically and pathologically hypertrophic rats, inflammation-related genes (such as pancreatitis-associated proteins and arachidonic acid 12 lipoxygenase) increased in the pathological process, but not in physiological hypertrophy (Kong et al., 2005). At the molecular level, the up-regulated expression of Rho kinase promoted inflammation. Fasudil is an inhibitor of Rho kinase. It was found that fasudil could significantly reduce the left ventricular dysfunction of pathological myocardial hypertrophy caused by partial aortic constriction (PAAC), but has no significant regulatory effect on left ventricular hypertrophy induced by chronic swimming training (CST). These results suggest that Rho kinase is involved in PAAC-induced pathological myocardial hypertrophy (Balakumar and Singh, 2006). However, it is not ruled out that the regulatory effect of Rho kinase on physiological hypertrophy is not obvious due to the down-regulation of immune pathway in physiological cardiac hypertrophy induced by CST. It remains further explored whether the down-regulation of this immune pathway affects the energy metabolism of the myocardium.

## Conclusions

Metabolic remodeling is closely related to the occurrence and development of cardiac hypertrophy and other heart diseases. Exploring the energy metabolism mechanism of cardiomyocytes may lead to new therapeutic targets, which will be helpful to design new effective methods and strategies for the treatment of heart failure (Bøgh et al., 2020; Makrecka-Kuka et al., 2020; Zuurbier et al., 2020). As we all know, physical exercise has a protective effect on the heart, and can even partially compensate for heart damage and improve heart function. Combined with the metabolic adaptation pathway observed in the studies of myocardial physiological hypertrophy induced by exercise, we can further explore how exercise-induced metabolic adaptation coordinate cell signals and gene expression, which may be helpful to guide exercise training scientifically, maximize the benefits of exercise, improve heart health, and develop new treatment strategies for the treatment of heart failure caused by various complex reasons and aging in the future.

## Author Contributions

KX and ZQ contributed equally to this work. KX wrote the manuscript. ZQ and HZ analyzed concerned literatures. XL and ZQ revised the manuscript. All authors agree to be accountable for the content of the work.

## Funding

This study was sponsored by the National Natural Science Foundation of China (81773726 to XL), National Science and Technology Major Project (2018ZX09711002-003-015 to XL), Shanghai Sailing Program (19YF1459500 to ZQ) and Science and Technology Innovation Action Plan Project (19401900100 to XL and 19431901400 to ZQ).

## Conflict of Interest

The authors declare that the research was conducted in the absence of any commercial or financial relationships that could be construed as a potential conflict of interest.

## Abbreviations

ATGL, adipose triglyceride lipase; GPCRs, G-protein-coupled receptors; FAT/CD36, fatty acid translocases; FATP, fatty acid transporters; FA-CoA, acetyl CoA; ACC, Acetyl coenzyme A carboxylase; PPARα/β, peroxisome proliferators-activated receptor α/β; PGC-1α/β, Peroxisome proliferator-activated receptor-γ coactivator-1α/β; OXPHOS, oxidative phosphorylation; ETC, electron transfer chain; FAO, fatty acid oxidation; ERR, estrogen-related receptors; NRF-1, nuclear receptor factor 1; eNOS, endothelial nitric oxide synthase; LCFA, long-chain fatty acids; PI3K, phosphatidylinositol 3 kinase; IRS, insulin receptor substrate; IGF-1R, insulin-like growth factor-1 receptor; PKC-α, protein kinase C-α; O-GlcNAcylization, O-junction of monosaccharide β-N-acetyl-glucosamine; HDAC, histone deacetylase; MCU, mitochondrial calcium uniporter; VEGFs, vascular endothelial growth factors; CST, chronic swimming training; PAAC, partial aortic constriction; AMPK, Adenosine 5’-monophosphate (AMP)-activated protein kinase.
